# Importance of Electrostatic Interactions in the Association of Intrinsically Disordered Histone Chaperone Chz1 and Histone H2A.Z-H2B

**DOI:** 10.1371/journal.pcbi.1002608

**Published:** 2012-07-12

**Authors:** Xiakun Chu, Yong Wang, Linfeng Gan, Yawen Bai, Wei Han, Erkang Wang, Jin Wang

**Affiliations:** 1 College of Physics, Jilin University, Changchun, Jilin, P.R. China; 2 State Key Laboratory of Electroanalytical Chemistry, Changchun Institute of Applied Chemistry, Chinese Academy of Sciences, Changchun, Jilin, P.R. China; 3 Laboratory of Biochemistry and Molecular Biology, National Cancer Institute, Bethesda, Maryland, United States of America; 4 Department of Chemistry and Physics, State University of New York at Stony Brook, Stony Brook, New York, United States of America; University of Missouri, United States of America

## Abstract

Histone chaperones facilitate assembly and disassembly of nucleosomes. Understanding the process of how histone chaperones associate and dissociate from the histones can help clarify their roles in chromosome metabolism. Some histone chaperones are intrinsically disordered proteins (IDPs). Recent studies of IDPs revealed that the recognition of the biomolecules is realized by the flexibility and dynamics, challenging the century-old structure-function paradigm. Here we investigate the binding between intrinsically disordered chaperone Chz1 and histone variant H2A.Z-H2B by developing a structure-based coarse-grained model, in which Debye-Hückel model is implemented for describing electrostatic interactions due to highly charged characteristic of Chz1 and H2A.Z-H2B. We find that major structural changes of Chz1 only occur after the rate-limiting electrostatic dominant transition state and Chz1 undergoes folding coupled binding through two parallel pathways. Interestingly, although the electrostatic interactions stabilize bound complex and facilitate the recognition at first stage, the rate for formation of the complex is not always accelerated due to slow escape of conformations with non-native electrostatic interactions at low salt concentrations. Our studies provide an ionic-strength-controlled binding/folding mechanism, leading to a cooperative mechanism of “local collapse or trapping” and “fly-casting” together and a new understanding of the roles of electrostatic interactions in IDPs' binding.

## Author Summary

Histone chaperones facilitate the assembly and disassembly of nucleosome by interacting with the corresponding histones or histone variants. As the biomolecules in nucleosome are highly charged, electrostatic interactions are particularly important in these processes. The experiments have explored that the histone chaperon Chz1 as an intrinsically disordered protein (IDP) can fold by binding to its histone variants H2A.Z-H2B. Here, we developed a molecular simulation program that treated electrostatic interactions with Debye-Hückel model to study the mechanism of the association. We found that the inter-chain electrostatic interactions facilitate the coupled folding and binding transitions, consistent with the kinetic experiments and microscopic structural perspectives. Furthermore, we show that the intra-chain electrostatic interactions collapse Chz1 and slow the binding rate. The collapsed structure in IDPs caused by intra-chain electrostatic interactions has been widely found in experiments and the effect in binding is well studied in our work. Our theoretical approach shed new light on the role of electrostatics on inter-chain and intra-chain interactions for IDPs' binding and is applicable to the binding/folding of other IDPs to their targets.

## Introduction

Nucleosome, the fundamental repeating structural unit of chromatin, is comprised of two superhelical turns of DNA (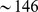 base pairs) wound 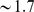 times around an octamer of histone proteins (H2A, H2B, H3, H4) or their variants [1]–[4]. Histone chaperones prevent histones from aggregating on DNA by blocking the DNA-binding sites on histones [5]–[7], and play essential roles in the assembly and disassembly of the nucleosome [8]–[11]. The histone proteins are highly positively charged and usually associated with their binding partners, such as DNA and histone chaperones, through electrostatic interactions [12]. However, little is known about the processes as how histone chaperones associate and dissociate from the histones, which could be closely related to how histone chaperones deliver the histones to the target molecules. Because of the oppositely charged characteristic between histone chaperones and histones, the electrostatic interactions rather than hydrophobic interactions are supposed to highly participate in these molecular events. Moreover, some histone chaperones are intrinsically disordered proteins (IDPs) [9], indicating that the association and dissociation are also coupled with folding and unfolding of polypeptide chains. The studies of IDPs have put forward a new dynamics-function paradigm for biomolecular recognition [13], [14].

**Figure 1:**
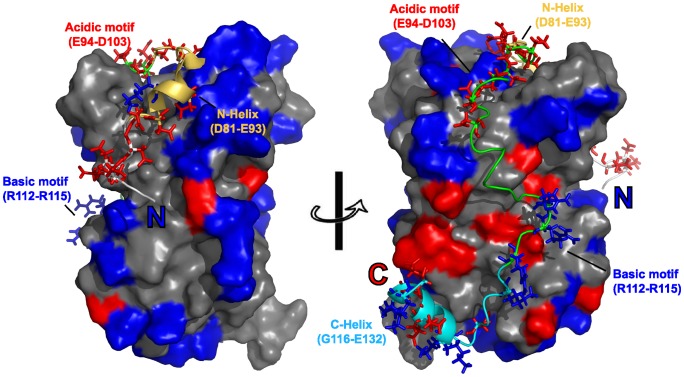
Charge distribution in the structure of the Chz.core-H2A.Z-H2B complex. Histones are in surface model. Chz.core is shown in cartoon representation. Residue Lys and Arg are colored blue; Residue Glu and Asp are colored red. The charged residues in Chz.core is shown in sticks representation. The negatively charged N-terminus of Chz.core and bipolar charged Chz motif form wide electrostatic interaction distributions with the histones, while the electrostatic interaction between C-terminus of Chz.core and the histones is not prevalent. The color representation for Chz.core: yellow, N-terminal helix (residues 81–93); green, Chz motif (residues 94–115); cyan, C-terminal helix (residues 116–132).

Chz1 (159 amino acids) is the chaperone of histone variant H2A.Z-H2B. Its function involves the delivery of H2A.Z-H2B to the SWR1 complex that catalyzes the exchange of H2A-H2B in the canonical nucleosome with H2A.Z-H2B in an ATP-dependent manner [15]. Chz1 is an IDP and binds to H2A.Z-H2B using its middle region (residues 71–132), termed Chz.core [15]. Upon binding to H2A.Z-H2B, Chz.core forms two short helical structures at the N- and C-terminal regions and a long irregular loop in the middle (Figure 1) [16]. In contrast, the conformation of H2A.Z-H2B in the Chz.core-H2A.Z-H2B complex is essentially the same as the free H2A.Z-H2B [17]. The N-terminal region of Chz.core (residues 71–93) is largely negatively charged and interacts with the positively charged region in the H2A.Z-H2B while the region near the C-terminus has three positively charged arginine residues and interacts with several acidic residues in the H2A.Z-H2B. The bipolar charged Chz motif (residues 94–115) forms interactions with H2A.Z-H2B through complementary electrostatic forces. The NMR structure of Chz.core complexed with H2A.Z-H2B shows that the complex seems to be mainly stabilized through broad electrostatic rather than hydrophobic interactions. These structural features lead to the observation that Chz.core has a higher association rate than the diffusion limit, suggesting that the association process is accelerated by the electrostatic interactions [17].

To better understand the folding/binding of Chz.core to H2A.Z-H2B, we performed theoretical investigations on the underlying mechanisms from thermodynamic, kinetic and microscopic structural perspectives. We developed a structure-based coarse-grained model [18], [19] to simulate the formation of the Chz.core-H2A.Z-H2B complex. In particular, we implemented 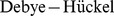 model to describe the electrostatic interactions. We observed two parallel binding/folding pathways in our simulations. By calculating the reaction rates, we found that the electrostatic interactions serve as the “steering forces” to facilitate the association, coincident with the NMR spectroscopy experiments. However, we found that the electrostatic interactions did not always accelerate the formation rate of the complex. Under low salt conditions, non-native electrostatic interactions transiently trapped Chz.core in the ensemble of collapsed structures slowed down folding/binding. It is worth noting that the Chz1 studied in our simulations only consider the Chz.core (residues 71–132), which is found to be responsible for the inter-chain interactions to stabilize the complex Chz1-H2A.Z-H2B [15]. It is consistent with the NMR experiment [17].

## Results

### Chz.core undergoes disorder-to-order transition upon binding

**Figure 2:**
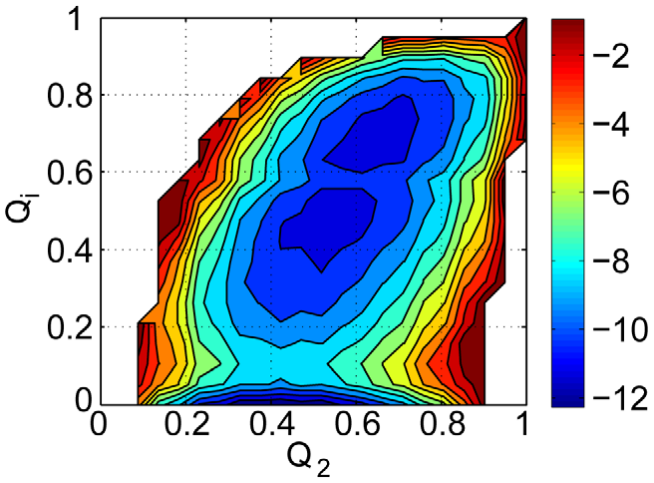
Free energy landscape calculated from the thermodynamic simulation. Free energy surfaces are plotted as a function of 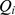, 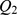. 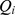, 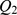 represent the structural similarity of inter-chain binding and intra-chain folding of Chz.core to the bound state in the binding process. The free energy profiles provide a global mechanism of the association. Free energy is in the unit of 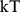.

We plotted free energy along 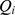 and 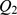 (Figure 2) to illustrate how the binding/folding process happens. 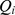 is the fraction of native contacts between H2A.Z-H2B and Chz.core, 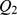 is the fraction of native contacts for folding of Chz.core. In unbound state, Chz.core comprises of a large number of unfolding conformations, consistent with a typical IDP. In contrast, the structure of H2A.Z-H2B remained folded and almost unchanged in both free and bound states. These structural features are in accordance with the NMR spectroscopy experiments, guaranteing the validity of our model. The free energy profile shows a typical 3-state binding transition with the first free energy barrier of 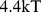 at 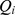 of 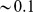, indicating that the initial recognition of Chz.core by the histone variant occurs very early. The second lower free energy barrier at 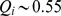 separates the intermediate states and the native bound state. The highest free energy region with 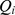 of 0.06–0.13 is taken as the initial binding transition state ensemble. In the initial transition state ensemble, some local regions of the conformations of Chz.core make native contacts with the histone variant while Chz.core remains largely disordered. This implies that significant binding happens when the Chz.core is partially disordered before complete folding. This is different from the conventional scenario of folding first with the structure formation of individual partners and then binding last. In our simulations, the coupled folding and binding of Chz.core occurs mainly after this transition state.

### The rate-limiting transition state occurs early

To better characterize the structural ensemble of the first transition state and identify the key residues for the recognition between Chz.core and H2A.Z-H2B, we calculated the 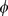 value for each residue in Chz.core. 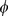 value has been used in protein folding for revealing the native interactions in the transition state [20]. The 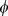 value here only counts native binding contacts and has a simple expression: 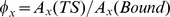. 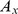 is the number of native contacts between Chz.core residue x and H2A.Z-H2B residues in the respective state. For 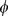 value calculation, we selected all of the conformations with 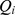 in the range of 0.06–0.13 from the simulation. We found that all 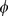 values are smaller than 0.5 (see Figure S1 in Text S1), indicating that none of the residues in Chz.core are well-ordered. In particular, the 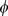 values at the C-terminal region (residues 116–132) approach to 0, suggesting that it has little native contacts with H2A.Z-H2B in the initial binding transition state.

**Figure 3:**
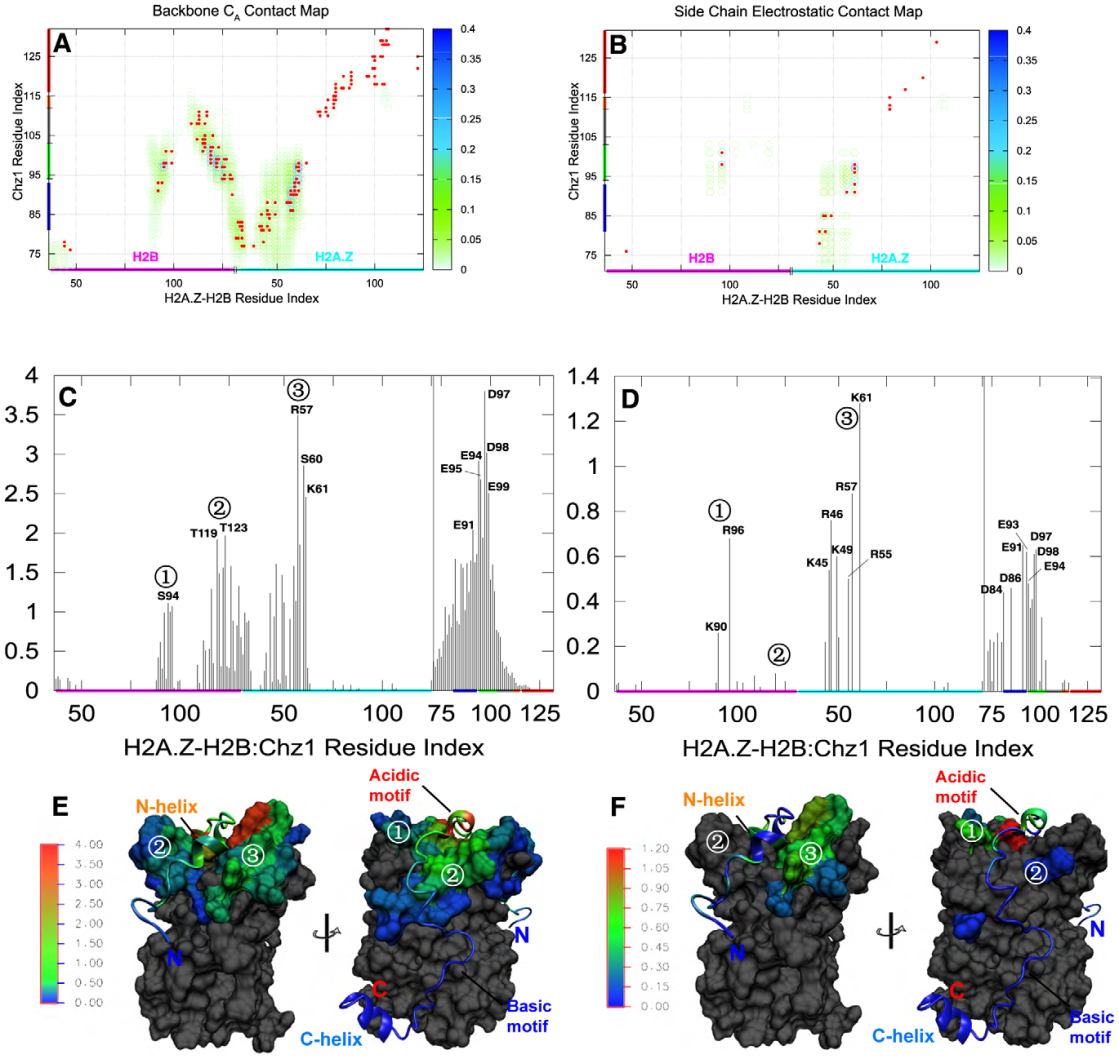
Contacts in the transition state. (A) The 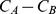 contact map. (B) The side chain contact map for oppositely charged residues. The circles with gradational color changes represent the probability of contact existing in the transition state. Red points represent the contacts existed in the native structure. The average contact number of each residue formed by 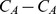 and oppositely charged side chain interactions are represented in (C) and (D) and illustrated in (E) and (F) respectively. Different part of Chz.core are in different color representations in (A), (B), (C), (D): Blue, N-terminal helix; green, the acidic motif (residues 94–103); grey, the neutral motif (residues 104–111); orange, the basic motif (residues 112–115); red, C-terminal helix. The H2B and H2A.Z with residue sequence are marked on X-axis. The three “hot spot” regions of H2A.Z-H2B: (1) N88-T100 of H2B, (2) E109-A131 of H2B and Q29-A33 of H2A.Z,(3) Q38-A53 of H2A.Z are shown with numbers. In (E) and (F), the structures of bound state are color-coded according to the values of average contact number of residues in transition state. For a better visualization, the residues on H2A.Z-H2B which do not have inter-chain contacts are shown in grey.

Since 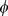 values lacking the non-native contacts may not yield an accurate interaction map of the transition states, we introduced a cut-off contact map to count non-native contacts and calculated the contact maps of both 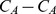 and 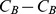, representing backbone–backbone and side chain–side chain interactions respectively. In the transition state, the N-terminal region and the acidic motif (residues 94–103) of Chz.core form wide contacts with the histones, while the C-terminal region has little inter-chain interactions (Figure 3A). Furthermore, Figure 3B shows that the N-helix (residues 81–93) and the acidic motif of Chz.core, carrying many negatively charged residues (E91 to D103), form wide electrostatic interactions with the positively charged residues: K89, K90 in H2B and R55, R57, K61 in H2A.Z. Meanwhile, the negatively charged region (E73 to D81) located at the N-terminal region of Chz.core forms electrostatic interactions with the positively charged region (R43 to K61) of H2A.Z. Notably, most of the contacts are referred to non-native contacts. Those non-native interactions can act as “steering forces” in the early binding process to facilitate the association [21].

Next, we calculated the probability of contact formation and the average number of contacts for each residue (Figure 3C–F). We found that the acidic motif and residue E91 of the N-helix of Chz1 have the largest number of contact residues, which are all negatively charged. In contrast, the “hot spot” residues in histone variant can be roughly divided into 3 regions: (1) residues from N88 to T100 in H2B; (2) residues from E109 to A131 in H2B and from Q29 to A33 in H2A.Z; (3) residues from Q38 to A63 in H2A.Z. By examining the contact map for the side chain electrostatic interactions, as residues in (1) and (3) of H2A.Z-H2B form abundant electrostatic interactions with Chz.core, it appears that association of those regions in H2A.Z-H2B are charge-oriented. On the other hands, the binding of Chz.core seems to start at N-terminal region and the acidic motif, which is highly charged. In conclusion, the electrostatic interactions appears to have strongly affected the transition state.

### Chz.core passes through two parallel binding pathways

**Figure 4:**
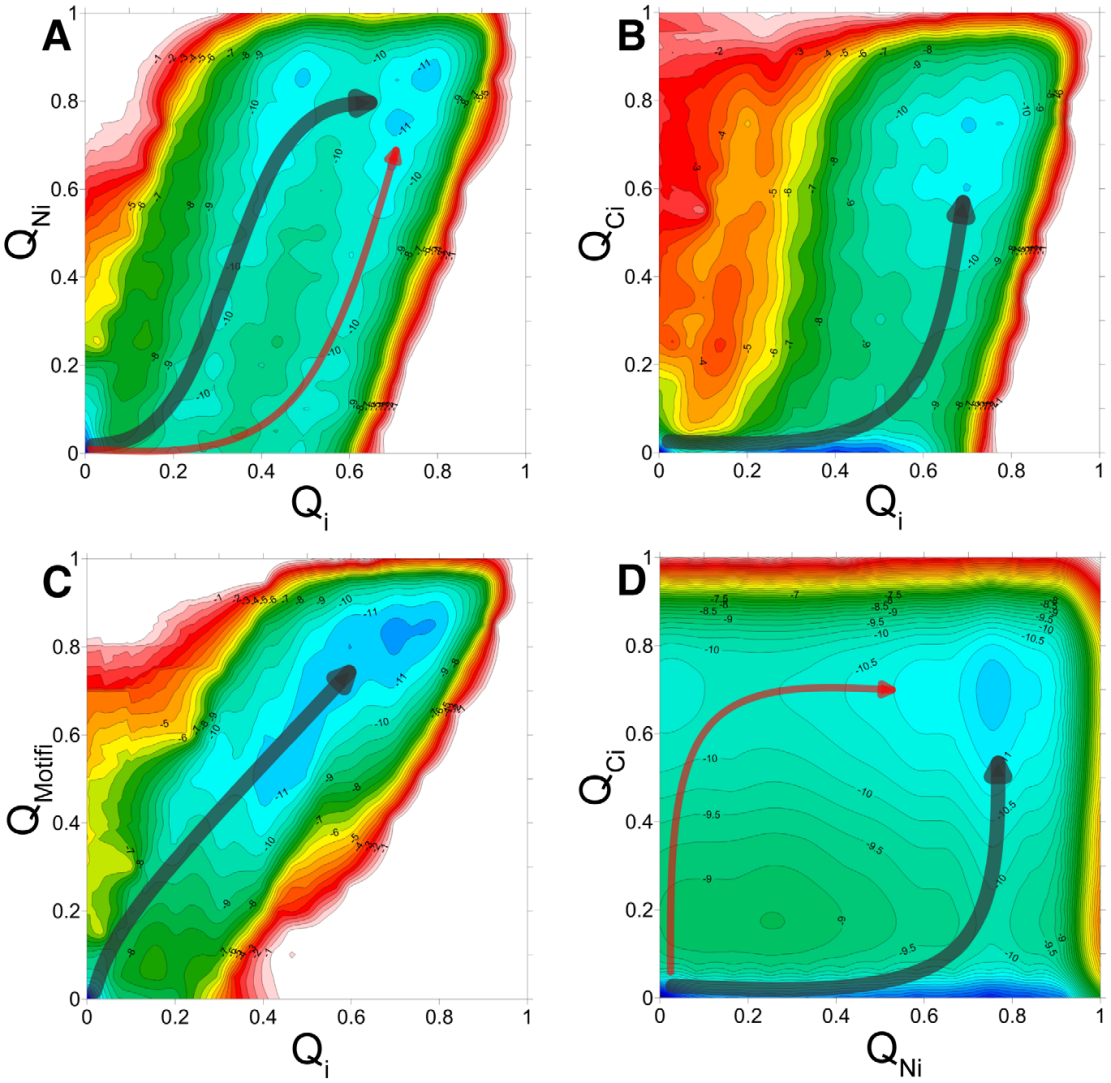
Free energy landscape. 2D-free energy profiles as a function of (A) 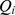 and 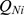, (B) 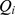 and 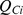, (C) 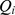 and 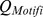, (D) 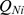 and 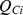. 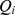 measures the global degree of binding process. 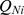, 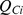 and 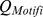 measure the degree of binding of N-helix, C-helix, Chz motif in Chz.core to the histones. There are two distinct binding pathways and intermediate states in (A), (D), only one pathway formed one intermediate state in (B) and only one pathway without intermediate state in (C). Free energy is in the unit of 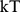.

To characterize the binding pathway of N-helix, C-helix and Chz motif of Chz.core in detail, we plotted a series of 2-D free energy landscape as a function of 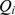, 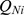, 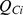 and 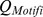 (Figure 4). 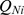, 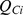, 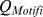 are the fractions of inter-chain native contacts between the regions of N-helix, C-helix, Chz motif in Chz.core and H2A.Z-H2B, respectively. The 3 regions of Chz.core bind to the histones through different patterns: The binding of N-helix takes off at 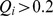 (Figure 4A), when there is not much binding for other regions of Chz.core; the binding of C-helix is unique and can only occur at 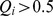 (Figure 4B), when N-helix or Chz motif have already certain degrees of binding; the free energy profile of binding of Chz motif shows no intermediate state (Figure 4C), indicating that the binding of Chz motif is highly coupled with the binding of the whole chain.

**Figure 5:**
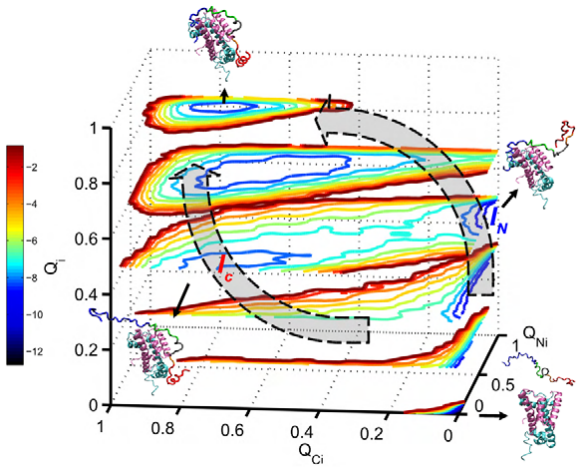
Parallel binding pathways. 3D-free energy lanscape as a function of 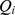, 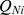, 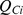. 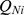, 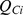 represent similarity of binding between N-helix and C-helix of Chz.core and histones to the bound state. There are two binding pathways connecting the unbound state and bound state. The two pathways go through intermediate 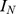 and intermediate 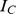. 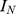 is more populated than 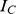 as it shows a deeper free energy minima. The representative structures of bound state, intermediate 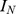, 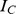 and unbound state are shown with color representation for Chz.core: Blue, N-terminal region; green, the acidic motif; grey, the neutral motif; orange, the basic motif; red, C-terminal helix. The missing backbone atoms of histones in coarse grained model are added for a better visualization. Free energy is in the unit of 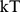.

The binding of the two helices to the histones are highly decoupled since there is no binding pathways along the diagonal line, suggesting there are two parallel binding pathways (Figure 4D). Therefore, we plotted the 3-D free energy landscape as a function of 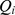, 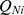, 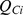 to investigate the binding pathways (Figure 5). We found that there are two binding intermediates, one for each pathway, as indicated by the minimum points on the free energy profile. By analyzing the equilibrium trajectories, we calculated the population of the two intermediates: 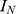 (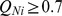 and 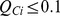) and 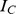 (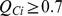 and 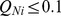) occupy 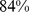 and 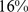 of the total population of intermediates, respectively. The results are likely due to the fact that the N-terminal region of Chz.core can form much more electrostatic interactions with H2A.Z-H2B than the C-terminal region does. So we can conclude that the long-range electrostatic interaction seems to be the driving force for the binding process, leading to an increased capture radius for searching the target. After the initial recognition, the partly bound intermediates are stabilized by the short-range electrostatic interactions.

To gain further insights into the structures of the two intermediate states, we investigated the native contact map in the two intermediate ensembles (see Figure S2 in Text S1). We found that the folding and binding of Chz.core at both intermediate states are highly coupled. In 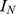, Chz.core binds to the histones by the N-terminal region and the acidic region of the Chz motif in their folded conformation. In 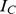, Chz.core binds to the histones by the folded C-helix and the Chz motif. It is interesting to point out that the conformations of the Chz motif in the two intermediates are not the same. The result implies that in 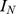, the Chz.core is more free and flexible. In addition, we found that the barrier height between intermediates 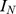, 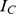 and the bound state are very low (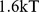 and 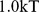 respectively). Such low barrier and the close value of 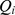 between intermediate 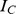 and bound state make the two parallel binding pathways mixed and the intermediate 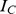 unobservable in Figure 4B. We also found that binding of Chz motif both occurs on the two parallel pathways, leading to only one binding pathway shown in Figure 4C.

As our simulations are carried at a higher temperature near to the binding transition temperature, the intermediates observed in our simulation are not more populated than the unfolded or disassociated state under equilibrium conditions. Decreasing the temperature to the experimental temperature will bias the free energy basin to the bound state (see Figure S5 in Text S1) and the low barriers from intermediate state to bound state make the intermediates are not detectable in the NMR relaxation dispersion experiment [17]. Meanwhile, as the barrier height in the range of thermal motion (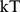) will be easily crossed, the intermediate 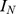 and 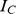 are not very thermodynamically stable and will overcome the barriers quickly to form the bound state in the binding process. In addition, the barriers of forming intermediate 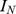 and 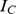 from unbound state are 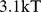 and 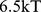, indicating that rate-limiting steps for the two parallel binding pathway are forming the intermediates.

### The kinetics is dependent on the salt concentration

**Figure 6:**
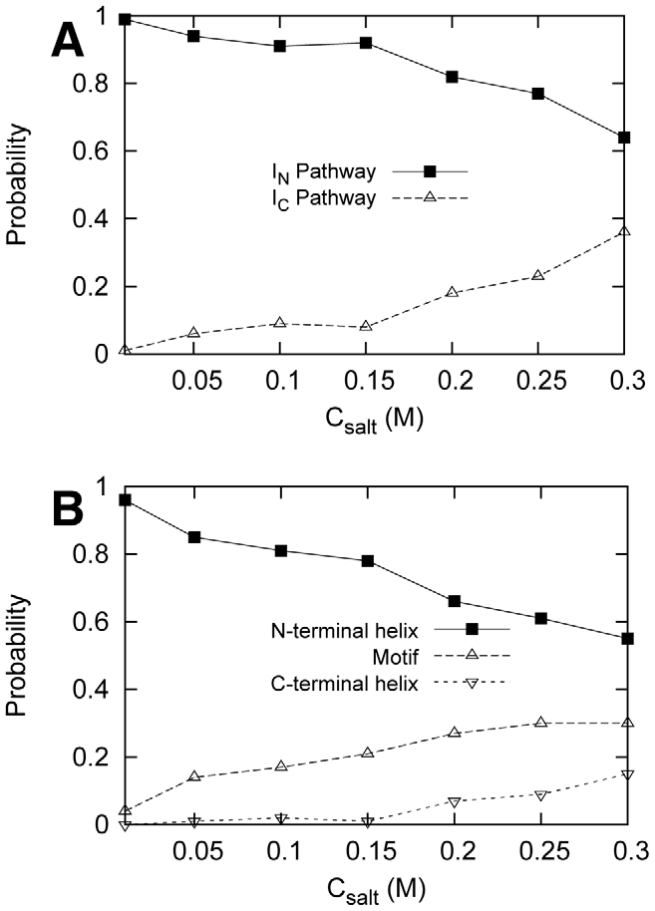
Distribution of binding pathway at different salt concentrations. Probability for (A) the two parallel binding pathways and (B) the first region of Chz.core to bind for N-helix, Chz motif, C-helix. N-helix are plotted with solid line while the motif and C-terminal helix are plotted with dotted line. We define the binding of the region completes when the corresponding fraction of native binding contacts exceeds 0.8.

To quantify the role of the electrostatic interactions in the binding process, we performed simulations at 7 different salt concentrations and a lower temperature than transition 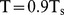. By investigating the kinetic trajectories, we also observed the two parallel binding pathways consistent with the thermodynamic simulations. In addition, we found that the weight of the two parallel binding pathway is modulated by the salt concentrations (Figure 6A). The population of 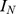 binding pathway increases when the salt concentration decreases, consistent with the conclusion that electrostatic interactions are the driving force for the binding of the N-terminal helix of Chz.core to the target. To explore how the electrostatic interactions affect the binding of N-helix, C-helix and the Chz motif, which have different charge distributions, we calculated the probability of the three regions to be the first to recognize the histones. We found that decreasing salt concentration leads to an increased probability for N-helix and decreased probability for the Chz motif and C-helix (Figure 6B).

**Figure 7:**
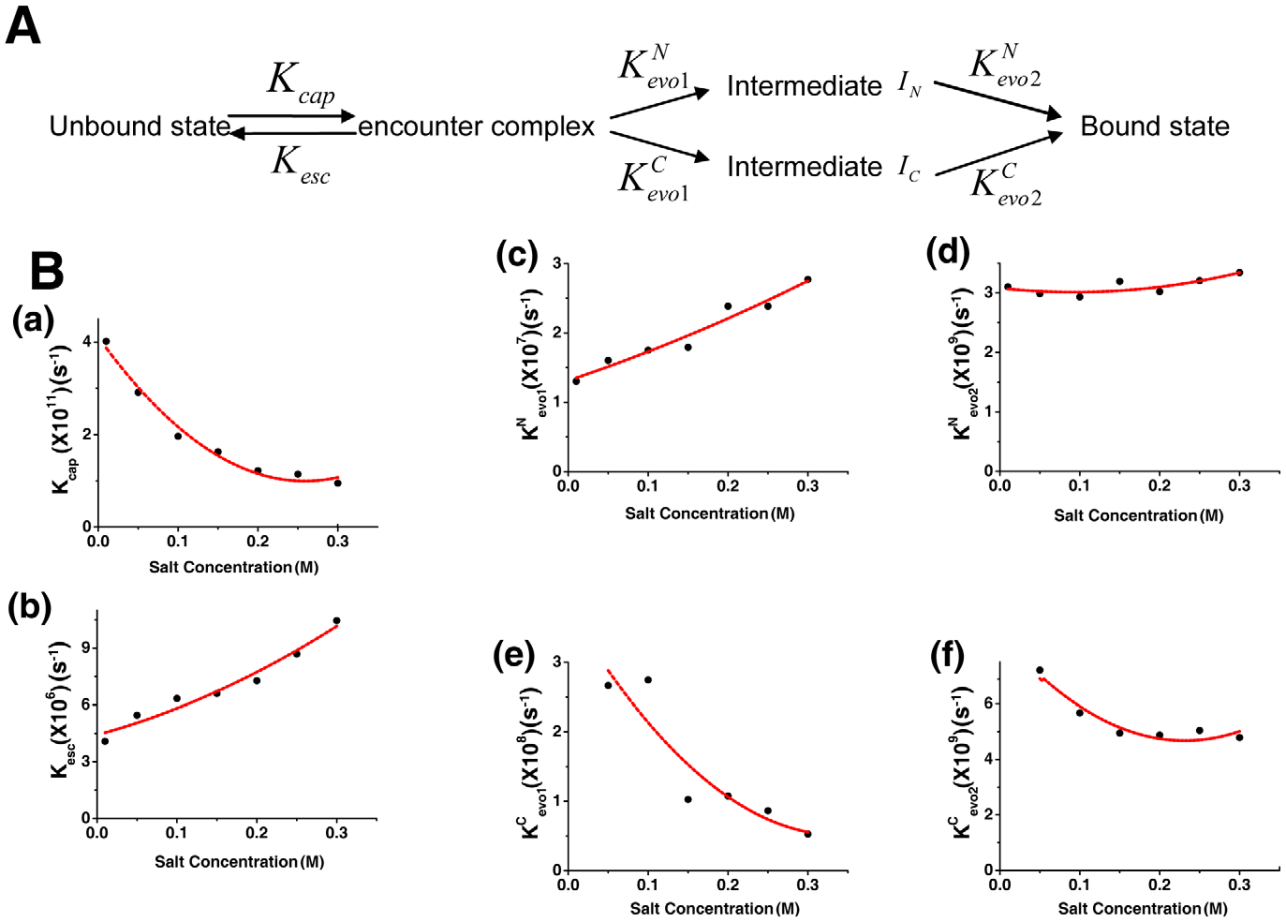
The binding rates are modulated by the salt concentration. (A) The binding process is divided into four steps: encounter, escape, evolution to the Intermediate states, and folding to the native state. 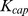 and 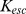 are the rates from unbound states to encounter states and from encounter states to unbound states, respectively. The last two steps can be dissected into two parallel pathways, forming two different intermediate states. 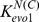 and 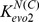 are the evolving rates from encounter states to intermediate states 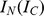 and from intermediate states 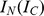 to bound states, respectively. (B) The 6 typical rates at different salt concentrations. (a,b) The rate 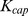 and 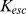 are shared by the two parallel pathways. The evolution rate in (c,d) 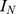 binding pathway and (e,f) 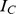 binding pathway shows different behavior as the salt concentration changes. All the rates are calculated by using transition number (N) and mean passage time (MPT). The dot lines are plotted to fit the grid data for a better visualization.

Because of the existence of the intermediate states, we dissected the association process into 4 steps: encounter, escape, evolve to intermediate states, and form the native state [22]. In the binding process, the two helices of Chz.core form different intermediates states, leading to different binding pathways with different binding rates (Figure 7A). All the 6 rates for describing the kinetics of the binding were calculated by using transition number (N) and mean passage time (MPT) in the binding trajectories with following equation (Figure 7B) [23]:
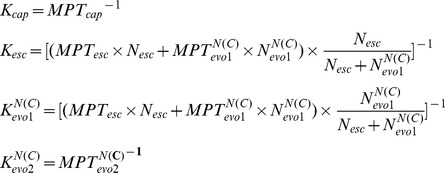



In the first capture step, the electrostatic interactions as “steering effect” significantly accelerate the recognition rate 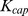 and decelerate the dissociation rate 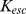. Then the binding is divided into two parallel pathways to form different intermediates. The rate of forming intermediate 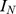 is slowed down, while the rate of forming intermediate 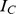 significantly increases as the salt concentration decreases. Based on the structural analysis of the two intermediates, the interactions between Chz.core and the histones in 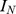 are mostly electrostatic. Thus it is surprising that the 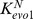 increases as electrostatic interactions decrease and the value is smaller than 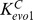 by 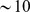 times. We found that there is a global structural rearrangement during the association from the free to the partly bound Chz.core (see subsection **Collapse slows binding**). The final step describes the evolution of the intermediate states to the bound states. For 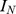 binding pathway, this evolution process corresponds to the binding of basic motif (residues 112–115) and the C-terminal region, these two regions are not highly controlled by electrostatic interactions, so the rate 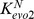 does not change with the different salt concentrations. On the other hand, on the 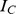 binding pathway, the evolution process from intermediate 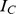 to the bound state corresponds to the binding of N-terminal region of Chz.core, which is highly charged. So the rate 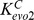 increases as the salt concentration decreases. The results that 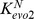 is smaller than 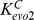 is coincident with the thermodynamic results, which shows a higher barrier to bound state for 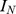 than 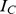 at 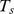. In addition, we found 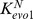 and 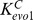 are much smaller than 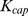, 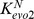, and 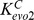, indicating that the the rate-limiting step are the evolution step from encounter step to intermediate state for the both binding pathways, consistent with the thermodynamic results. Although the kinetic simulations are performed at a lower temperature, this binding pattern is supposed not to be qualitatively changed from thermodynamic simulation [24].

### Collapse slows binding

**Figure 8:**
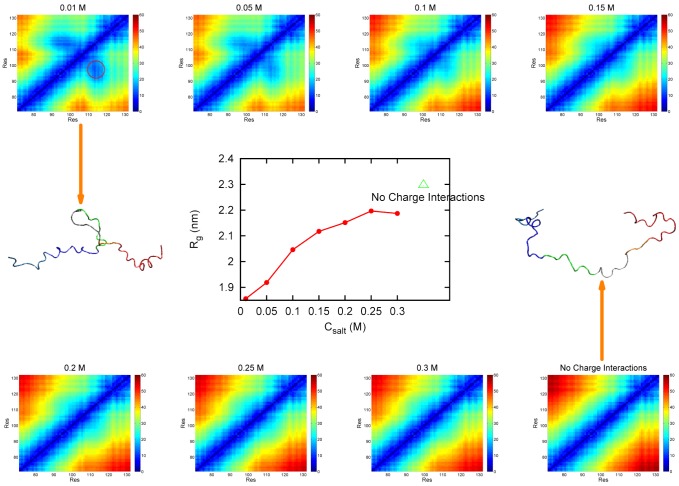
Side chain distance and radius of gyration 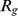 distributions in Chz.core at unbinding states along varying salt concentrations. The 8 pictures around the center panel show the side chain distance between two residues in Chz.core changes with different salt concentrations. The color goes from blue to red with increasing value of the distance. There is a tertiary collapsed structural region formed by residues 94–103 and residues 112–115 at low salt concentrations. The region is marked with red dashed circle in 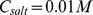 picture. The picture in the center represents the radius of gyration 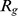 of Chz.core changes with different salt concentrations. Simulations in the absence of charge-charge interactions are also performed and the data are plotted as a benchmark. As salt concentration increases, 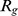 increases and the region of the collapsed structure becomes smaller and finally disappears. Two structures of Chz.core taken from the ensembles generated from 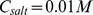 and in the absence of charge-charge interactions are intended to assist visualization of the development of the distance distribution map. Color representation for Chz.core: Blue, N-terminal region; green, the acidic motif; grey, the neutral motif; orange, the basic motif; red, C-terminal helix. The side chain distance is in the unit of 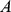, 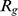 is in the unit of 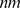.

In order to explain the abnormal relationship between 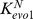 and the salt concentrations, we investigated the unbound states of Chz.core by looking into the structural differences as a function of salt concentrations. We used the distance of specific group of residues 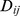 and radius of gyration 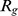 to detect the long-range interactions (Figure 8) since long-range contacts can exhibit a decrease in 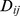 and 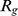 as compared with the idealized random coil ensemble [25]. We found that residues 94–103 and residues 112–115, corresponding to the acidic motif and the basic motif, are close in space at low salt concentrations. The formation of this local tertiary compact structure is due to the non-native electrostatic interactions between oppositely charged residues located at the two ends of the Chz motif, which disassociate as the ionic strength decreases.

**Figure 9:**
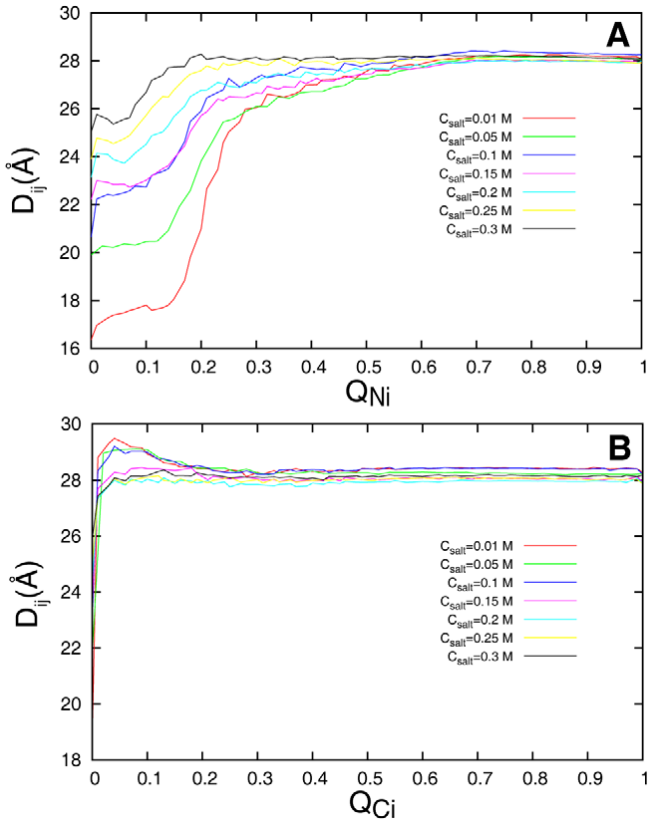
The evolution for the distance between the centroid of the side chains of the acidic motif and the basic motif. The values of the distance indicate the structural changes at reaction coordinate (A) 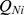 and (B) 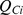. Different salt concentrations are represented by different colors.

In order to investigate how this local compact structure changes in the binding process, we plotted the evolution for the distance between the centroid of the acidic and basic motifs of Chz.core along 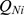 and 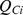 (Figure 9). 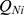 and 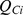 are used as reaction coordinates to describe the evolution step of the unbound states to the intermediate states on the 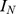 and 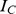 binding pathways respectively. We found that as the binding proceeds, the collapsed structure expands and becomes bound-like. However, this structural rearrangement appears in different steps on the two parallel binding pathways. On the 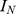 binding pathway, 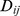 remains unbound-like when N-helix starts binding and has an abrupt change in 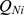 of 0.1–0.3 (Figure 9A), this local compact structure gets expanded and bound-like in the formation of the partly bound intermediates 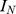. Unraveling the collapsed region consumes time. As the salt concentration decreases, this region of Chz.core becomes more collapsed at free states, so 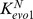 decreases. In contrast, on the 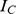 binding pathway, 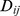 ascends acutely when 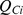 increases (Figure 9B), implying that the collapsed region has folded to its final bound structure in the beginning of the recognition. As a result, the rate of evolution from encounter complex to intermediate 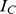 is not affected by salt concentration. Thus, it is very interesting to see that electrostatic interactions always accelerate rates on the 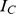 binding pathway. However, the electrostatic interactions decrease the rates on the 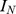 binding pathway when they are strong but increase the rates when they are weak.

## Discussion

In the thermodynamic and kinetic studies here, we provide a detailed binding-folding analysis on the formation of the Chz.core-H2A.Z-H2B complex with a structure-based coarse-grained model involving electrostatic interactions. We found that Chz.core, intrinsically unstructured in solution, folds upon binding to H2A.Z-H2B, consistent with the experimental observation [17]. The free energy profile shows Chz.core at the initial transition state remains unfolded resembling the unbound states, and then folds to the histone-bound structure through intermediate states as binding proceeds. Many IDPs seem to share such a common binding pattern, using the folded partner as a template to stabilize themselves rather than fold among themselves first [26]–[33].

Non-native interactions have been realized to play significant roles in protein folding and binding from experimental and simulation studies [26], [34]–[37]. The facilitating effect in association caused by non-native interactions are due to decreasing the transition barrier height [36], [37] or reducing the entropy on energy landscape at early stage [26]. In our studies, the contacts formed in initial binding transition states, are strongly oriented and positioned by the charge distribution, confirming that the electrostatic interaction is crucial in the recognition between the oppositely charged proteins [38]. Besides, the feature that the binding/folding pass through two parallel pathway is similar to that of pKID binding with KIX except for in the latter case, the dominant force is hydrophobic interaction [27], [28], [39], [40]. The close-range hydrophobic interactions are found to be the results of forming the intermediate states. Here, we demonstrated that the electrostatic interactions can also serve as the stabilizing forces in the forming of the partly bound complex [41], especially in binding of highly charged polypeptide chains.

IDPs in general can not fold to compact globular conformations in aqueous solutions because of their low hydrophobicity [13], [42]–[44]. The flexible conformation of IDPs makes them highly susceptible to the non-native electrostatic interactions, which can have a dramatic effect on folding energy landscape [45]. Net charges in IDPs can modulate the conformational space by changing the residue distance and radius of gyration [46]. Recently, a single-molecule fluorescence resonance energy transfer (FRET) spectroscopy study shows that the low hydrophobicity and highly charged property can make IDPs expand or collapse depending on ionic strength and concentration of denaturant [47], [48]. Consistent with the IDPs' experiments, we successfully observed the collapsed structure of Chz.core formed in unbound states in our work. Importantly, the effects of collapsed ensemble caused by varying salt concentrations in Chz.core for binding kinetics are studied in details. From our studies, the electrostatic interactions do not always accelerate the association through the whole folding/binding process [22], [49]. Instead, they have discrete effects on the two parallel binding pathways. The role of electrostatic interactions in this association can be interpreted from two different aspects: inter-chain electrostatic interactions enlarge the radii for fly-casting effect and facilitate binding [50], [51] while stabilizing non-native intra-chain interactions at low salt concentration lead to the collapse of Chz.core to form local compact conformations and decrease the rate of binding. From energy landscape theory, the binding landscape is referred to as a funnel [52], [53]. At the beginning of binding, two detached chains are at the top of the funnel with large entropy. The electrostatic inter-chain interactions act as a steering force to reduce the entropy to facilitate the recognition as “fly-casting” effect [50], while the stabilizing intra-chain non-native electrostatic interactions in Chz.core cause kinetic traps on the energy landscape and lead to the slowing down of the association as “local collapse or trapping” effect. Thus, the binding rate of Chz.core to H2A.Z-H2B is controlled by the balance of native and non-native electrostatic interactions [54]. The correlation between disordered structure and charged sequence in unfolded proteins implies that this scheme is common in the process of IDPs' binding.

In summary, we developed a structure based coarse-grained model that incorporates electrostatic interactions using 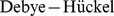 model for studying protein-protein interactions, including IDPs. We used the model to investigate the folding/binding mechanism of Chz.core in the formation of the Chz.core-H2A.Z-H2B complex, revealing that electrostatic interactions can accelerate folding by steering the association but can also cause non-native interactions that slow down folding. The findings here provide a new understanding the role of electrostatic interactions in IDPs' binding. Our approach is applicable to the binding/folding of other IDPs to their targets and can be extended to include the association of disordered regions in some chromatin factors with the nucleosome that has a broadly distributed negatively charged surface on DNA.

## Materials and Methods

From energy landscape theory, the folding/binding energy landscape of proteins should be minimally frustrated and has a shape of funnel [52], [53], [55]. The proteins' native topology can determine the mechanism of folding and binding. The structure-based model has been used to study the folding of monomeric proteins and the binding of oligomers, and can successfully reproduce the experimental results [18], [19]. Plain structure based model only considers the interactions existing in native structure mapping a much smoother energy landscape to ensure the simulation achievable. In order to study the effect of electrostatic interactions on this system, we developed a modified coarse-grained structure based model in which each amino acid was modeled by two beads except for glycine. The first bead (named 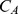 bead) belongs to the backbone of the protein chain, whereas another one (named 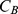 bead) represents the side chain by its centroid and is responsible for the physicochemical properties of the amino acid. Especially, we introduced the charged characterization into our 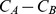 model to study the effect of electrostatic interactions on this system. The functional form of the forced field is given as a typical structure based model potential [56].
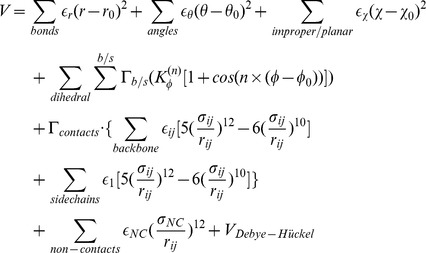



Where a few modifications are stated below: 1) the third term in this equation represents the chirality potential, which can maintain the correct chirality of the side chain. 2) The native contact map is built by Contacts of Structural Units (CSU) software [57]. An amino-acid-type dependent is used to describe the contacts between backbone and backbone [58]. The strength of each side chain–side chain interaction contributes the same weight to the potential. Backbone–side chain interaction is not introduced in the potential [59]. 3) The electrostatic interaction is represented by 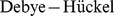 model, mimicking the effect of varying salt concentration:
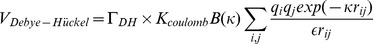




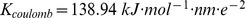
 is the electric conversion factor; 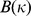 is the salt-dependent coefficient; 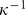 is the Debye screening length which is directly affected by salt concentration; 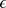 is dielectric constant and was set to 80 throughout the simulations. So the relationship between 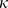 and salt concentration 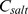 can be written explicitly: 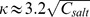. The exact physical meaning of 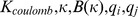 can be found here [35]. 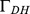 is the energy scaled coefficient which aims to make the total energy balanceable.

In our work, the parameters derive from the original folding/binding studies [18], [60], namely,
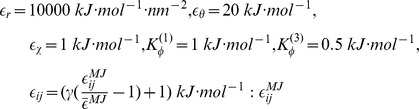
is the original MJ potential, 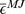 is the mean value of the entire set of MJ weights in this protein system, 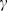 is a variable which can modulate the range of energetic heterogeneity; in the present work, 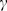 has been set to 1.0 corresponding to the “flavored model” [61]. 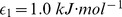 means that the contacts between side chains and side chains are weighted equally in the force field. In order to maintain the model energetic balanceable, we introduced two factors in front of dihedral energies and contact energies. 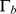, 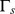 are chosen so that the ratio of backbone to side chain dihedral energy strength equals 2.0; 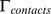 is set to make the ratio of total contact to total dihedral energy strength equals 2.0. In our reduced model, only Arg and Lys have a positive point charge and Asp and Glu have a negative point charge. The charges are placed on the 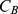 atoms mimicking the side chains. If the two side chains has already formed a salt bridge, its 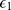 equals 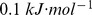, so that its total energetic contribution will be similar to the other contacts [51]. In simulation, we set 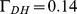, in this case if 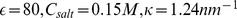, the DH potential for two opposite charges located at a distance of 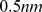 is equal to native contact energy 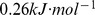.

In order to study how the electrostatic interactions influence the thermodynamic properties of the system, we performed a group of constant temperature simulations. The simulations were conducted at 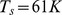 started from 40 different configures either dimeric or dissociative expecting to observe the most transitions in a limited simulation time. The total simulations were running 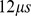 accumulating 396 binding transitions between unbound states and bound states to ensure the rationality for the thermodynamics analysis. The dynamics with electrostatic interactions was explored at a salt concentration of 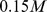 near to the physiological conditions.

In order to study how the electrostatic interactions influence the kinetic properties of the system, we simulated 100 trajectories at each salt concentration started from varying dissociative configures with different initial velocities. The dissociative configures comprised of folded histone variant H2A.Z-H2B and unbound histone chaperone Chz.core, were extracted from high temperature simulations. These kinetic simulations were done at a series of dilute solution in the range of 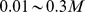 to guarantee the validity of the 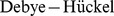 model. The temperature was set to 55 K (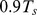). The first passage time (FPT) of certain region in Chz.core is calculated when the fraction of native binding contacts 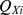 exceeds 0.8 at the first time, where “X” can be “N”, “Motif” and “C”, corresponding to the N-helix, Chz motif and C-helix of Chz.core, respectively. We calculated 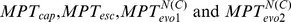, corresponding to the mean passage time from unbound states to encounter states, from encounter states to unbound states, from encounter states to intermediate states 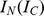 and from intermediate states 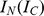 to bound states respectively, by averaging the 100 trajectories at each salt concentrations; and we also accumulated the corresponding numbers of transitions 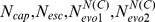 to calculate the 6 typical binding rates: 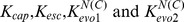 [23].

## Supporting Information

Text S1 Supporting information of electrostatic interactions in Chz1 binding to H2A.Z-H2B.
(PDF)
