# Supplementary material for: Importance of Electrostatic Interactions in the Association of Intrinsically Disordered Histone Chaperone Chz1 and Histone H2A.Z-H2B
Source: PLoS Comput Biol. 2012 Jul 12;8(7):e1002608. doi: 10.1371/journal.pcbi.1002608 (PMC3395605; doi:10.1371/journal.pcbi.1002608)
Supplement: Text S1 — Supporting information of electrostatic interactions in Chz1 binding to H2A.Z-H2B. (PDF) [file pcbi.1002608.s001.pdf]

Text S1

1 Simulation details

The CG-MD simulations used Langevin equation encoded in Gromacs software package to sample the conformation transition of the protein [1]. The two chains were placed at the center of a 50 nm × 50 nm × 50 nm cubic box. A strong harmonic potential was added if the distance between the center of mass of the two chains is farther than 3 nm corresponding to an effective protein concentration 14.7 mM [2]. The concentrations of histones and histone chaperons in chromatin are very high and the environment is very different from the common systems in vitro, which usually relate to dilute solution. It has been concluded that the binding affinity will be enhanced if the histone and histone chaperon are located at a crowding environment [3]. In silico, binding transitions are accelerated in the concentrated protein solution and the computational time are reduced [4].

2 Thermal factor in binding process

In order to see how the temperature influence the thermodynamic and kinetic properties of the system, we simulated 100 long time trajectories at 0.4 ∼ 1.1 T_s started from varying dissociative configures with different initial velocities. Each trajectory was performed 20 ns accumulating 2 μs at each temperature to ensure the thermodynamics analysis. Using the Weighted Histogram Analysis Method [5], we obtained the heat capacity and the thermodynamic properties at different temperatures. From Figure S3, we defined the transition temperature as $T_t = 60.3\ K$ at which the heat capacity is at its peak. We chose the temperature $T_s = 61\ K$ as our thermodynamic simulation temperature, so the equilibrium probabilities of unbound states and bound states are nearly equal.

We calculated the MFPT for binding of the 3 regions in Chz.core at different temperatures for a fixed salt concentration. We observed a typical V-shape binding curve for Chz.core (Figure S4A). The probability of the distribution of the two parallel binding pathway slightly changes with the temperature, and has a flat region at $0.6T_s \sim 0.9Ts$ (Figure S4B). We calculated the probability for the 3 regions of Chz.core to be the first to recognize the histones as a function of temperature (Figure S4C). Interestingly, we found that the probability for the N-terminal helix is the maximum when the MFPT of binding reaches its minimum at a temperature of $T = 0.6\ T_s$. In other words, if the N-terminal helix is the first region for the recognition, the rate of the overall binding will be the fastest. We note that previous theoretical calculations show that the MFPT is dependent on thermal instability, kinetic diffusion and local trapping states at varying temperatures [6]. Because the kinetics of N-terminal helix of Chz.core formation is strongly controlled by electrostatic interactions as concluded above, although the temperature changes the binding rate, the electrostatic interactions are the underlying dominant factor for the optimal binding rate.

Combining the trajectories at different temperatures, we obtained the thermodynamic properties of this system as shown by the free energy profile as a function of Q_i (Figure S5A). As expected, the native basin becomes deeper as temperature decreases, showing that the shape of free energy landscape strongly depends on the temperature. Meanwhile, the stability of the intermediates are strongly affected by the temperature as well. As the temperature decreases, the binding is strongly biased to the bound state and becomes “downhill” on the free energy landscape. Because the barrier for intermediate states transiting to bound states is low, we can approximately consider the binding process as a 2-state reaction for calculating dissociation constant K_D, the equilibrium can be described as:

$$H2A.Z\text{-}H2B + Chz1 \underset{k_{off}}{\overset{k_{on}}{\rightleftharpoons}} H2A.Z\text{-}H2B{:}Chz1$$

K_D for 2-state is calculated by:

$$K_D = \frac{[H2A.Z\text{-}H2B][Chz1]}{[H2A.Z\text{-}H2B{:}Chz1]} = \frac{k_{on}}{k_{off}}$$

We calculated the free energy along different Q_i, which produces the ratio of the free unbound molecule over the bound complex. Accordingly, we obtained K_D at different temperatures (Figure S5B). K_D decreases exponentially as temperature decreases. The experimentally observed K_D is 0.22 μM, suggesting that our thermodynamic simulations are performed at a higher temperature and protein concentration. A high but closed to transition temperature under concentrated protein condition is widely used in thermodynamics simulations for enhancing sampling more transitions between unbound states and bound states to save computational time. As there may be a critical concentration and temperature separating monomer and oligomer configuration phase, we argue that the underlying binding mechanism is not supposed to be qualitatively changed in a range of circumstances [7].

References

1. Hess B, Kutzner C, van der Spoel D, Lindahl E (2008) GROMACS 4: Algorithms for highly efficient, load-balanced, and scalable molecular simulation. J Chem Theory Comput 4: 435-447.

2. Bonomi M, Branduardi D, Bussi G, Camilloni C, Provasi D, et al. (2009) PLUMED: A portable plugin for free-energy calculations with molecular dynamics. Comput Phys Commun 180: 1961-1972.

3. Korolev N, Vorontsova OV, Nordenskiold L (2007) Physicochemical analysis of electrostatic foundation for DNA-protein interactions in chromatin transformations. Prog Biophys Mol Bio 95: 23-49.

4. Wang W, Xu WX, Levy Y, Trizac E, Wolynes PG (2009) Confinement effects on the kinetics and thermodynamics of protein dimerization. Proc Natl Acad Sci U S A 106: 5517-5522.

5. Kumar S, Bouzida D, Swendsen RH, Kollman PA, Rosenberg JM (1992) The weighted histogram analysis method for free-energy calculations on biomolecules .1. the method. J Comput Chem 13: 1011-1021.

6. Wang J (2006) Diffusion and single molecule dynamics on biomolecular interface binding energy landscape. Chem Phys Lett 418: 544-548.

7. Yang SC, Onuchic J, Levine H (2005) Protein self-assembly via domain swapping: The effect of intermolecular interactions and protein concentration. Biophys J 88: 198a-198a.

Table S1. The sequence information of Chz.core-H2A.Z-H2B.

Region	Residue information
H2B	Residues 37−131 of H2B
H2A.Z	Residues 29−125 of H2A.Z
Chz1	Residues 71−132 of Chz1
N-helix	Residues 81−93 of Chz1
N-terminal region	Residues 71−93 of Chz1
Chz motif	Residues 94−115 of Chz1
Acidic motif	Residues 94−103 of Chz1
Basic motif	Residues 112−115 of Chz1
C-helix	Residues 116−132 of Chz1

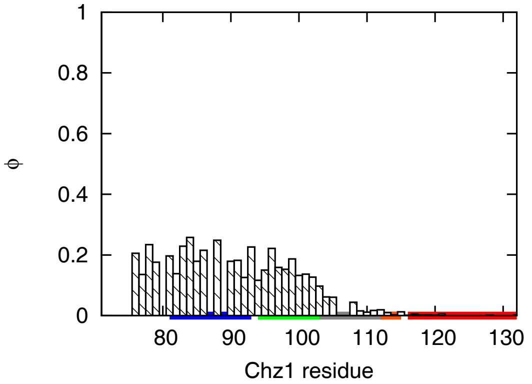

Figure S1. Binding ϕ values along the residue index of Chz.core. There is no residue with $\phi > 0$, indicating that all the residues of Chz.core in transition state are disordered. The color representation for Chz.core: blue, N-helix; green, acidic motif; grey, neutral motif; orange, basic motif; red, C-helix.

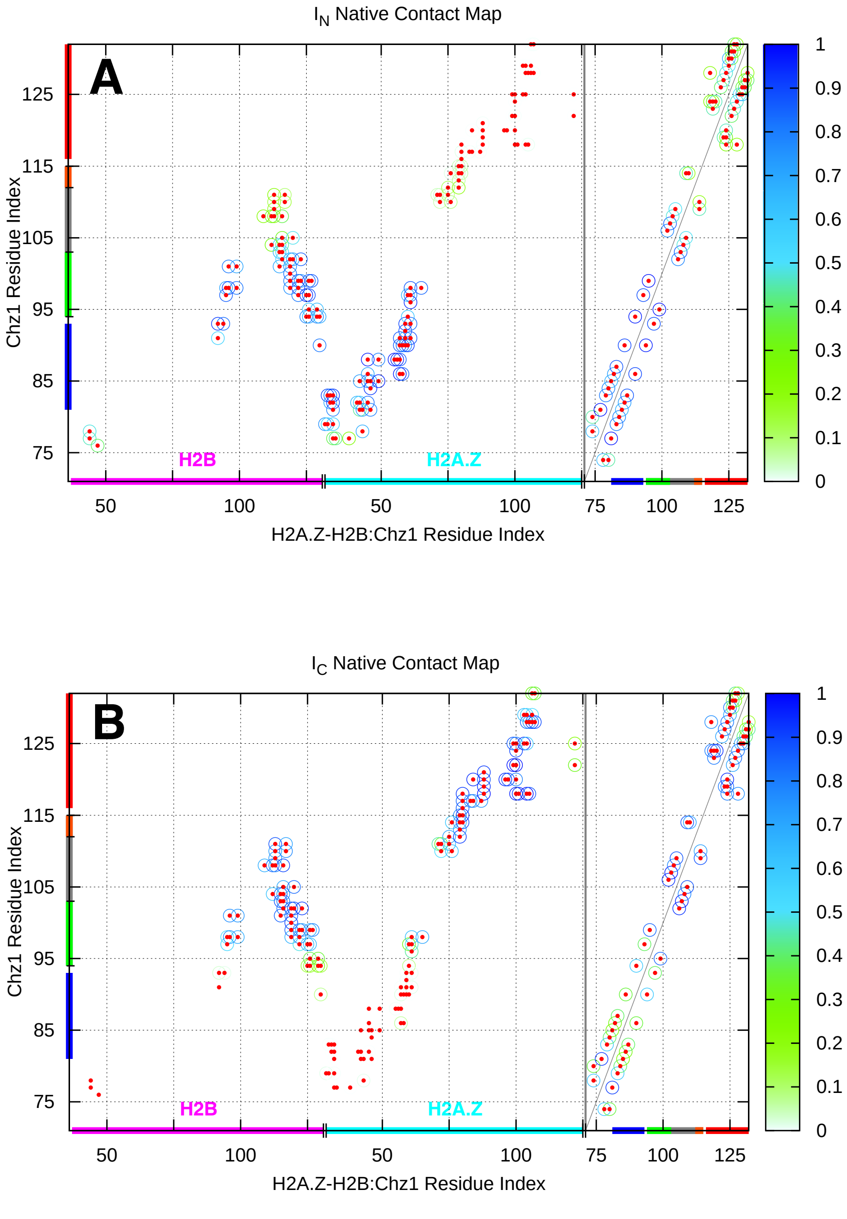

Figure S2. Native contact map in intermediates. Native contacts formed in the final bound state are plotted with the red solid circle, while the native contacts in the intermediate (A) I_N and (B) I_C are plotted with empty circle whose probability are represented by color shade. Different part of Chz.core are in different color representation. Blue, N-helix; green, acidic motif; grey, neutral motif; orange, basic motif; red, C-helix. The H2B and H2A.Z with residue sequence are marked on x-axis clearly.

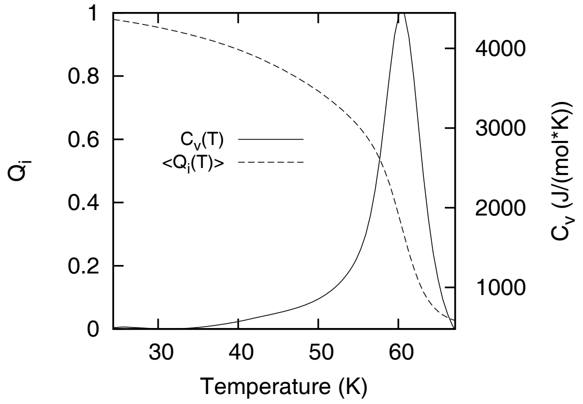

Figure S3. Heat capacity C_v and the average fraction of native binding contacts Q_i as a function of temperature. At the peak of heat capacity curve, the temperature are referred to as binding transition temperature with $T_t = 60.3\ K$. The $< Q_i >$ shows a monotonic decrease as temperature increases, the absolute value of the decreasing slope gets its maximum at T_t.

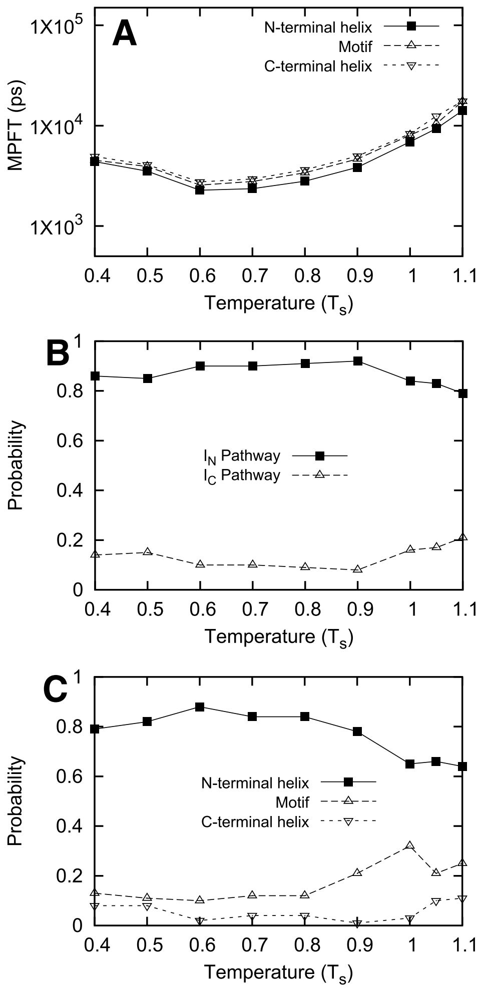

Figure S4. Distribution of binding pathway at different temperatures. (A) MFPT at different temperatures for N-helix, Chz motif, C-helix. (B) Probability for the two parallel binding pathways and (C) the first region of Chz.core to bind for N-helix, Chz motif, C-helix. MFPT is in the logarithm scale in the unit of ps, temperature is in the unit of T_s.

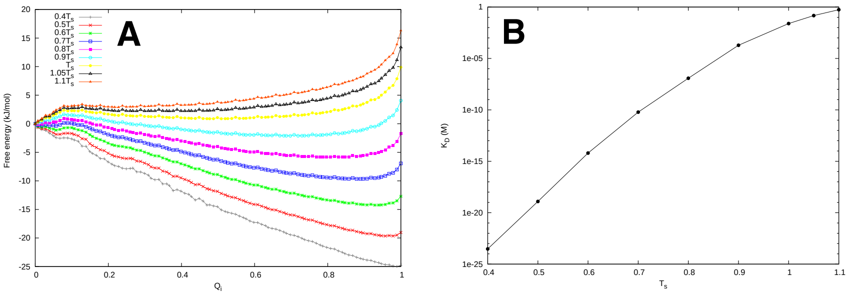

Figure S5. Free energy landscape and dissociation constant with different temperatures. (A) Free energy are plotted as a function of Q_i. (B) The dissociation constant K_D changes with temperature. The free energy is in the unit of kJ/mol.
